# Nomograms to predict survival after colorectal cancer resection without preoperative therapy

**DOI:** 10.1186/s12885-016-2684-4

**Published:** 2016-08-19

**Authors:** Zhen-yu Zhang, Qi-feng Luo, Xiao-wei Yin, Zhen-ling Dai, Shiva Basnet, Hai-yan Ge

**Affiliations:** 1 Department of Gastrointestinal Surgery, Shanghai East Hospital, Tongji University School of Medicine, Pudong New District, No. 150, Jimo Road, Shanghai, 200120 China; 2 Department of General Surgery, Qingpu Branch of Zhongshan Hospital, Fudan University, Shanghai, China

**Keywords:** Colorectal cancer, Nomogram, Cancer-specific survival, Overall survival, Decision curve analysis

## Abstract

**Background:**

The predictive accuracy of the American Joint Committee on Cancer (AJCC) stages of colorectal cancer (CRC) is mediocre. This study aimed to develop postoperative nomograms to predict cancer-specific survival (CSS) and overall survival (OS) after CRC resection without preoperative therapy.

**Methods:**

Eligible patients with stage I to IV CRC (*n* = 56072) diagnosed from 2004 to 2010 were selected from the Surveillance, Epidemiology, and End Results (SEER) database. The patients were allocated into training (*n* = 27,700), contemporary (*n* = 3158), and prospective (*n* = 25,214) validation cohorts. Clinically important variables were incorporated and selected using the Akaike information criterion in multivariate Cox regressions to derive nomograms with the training cohort. The performance of the nomograms was assessed and externally testified using the concordance index (c-index), bootstrap validation, calibration, time-dependent receiver-operating characteristic curves, Kaplan–Meier curves, mosaic plots, and decision curve analysis (DCA). Performance of the conventional AJCC stages was also compared with the nomograms using similar statistics.

**Results:**

The nomograms for CSS and OS shared common predictors: sex, age, race, marital status, preoperative carcinoembryonic antigen status, surgical extent, tumor size, location, histology, differentiation, infiltration depth, lymph node count, lymph node ratio, and metastasis. The c-indexes of the nomograms for CSS and OS were 0.816 (95 % CI 0.810–0.822) and 0.777 (95 % CI 0.772–0.782), respectively. Performance evaluations showed that the nomograms achieved considerable predictive accuracy, appreciable reliability, and significant clinical validity with wide practical threshold probabilities, while the results remained reproducible when applied to the validation cohorts. Additionally, model comparisons and DCA proved that the nomograms excelled in stratifying each AJCC stage into three significant prognostic subgroups, allowing for more robust risk classification with an improved net benefit.

**Conclusions:**

We propose two prognostic nomograms that exhibit improved predictive accuracy and net benefit for patients who have undergone CRC resection. The established nomograms are intended for risk assessment and selection of suitable patients who may benefit from adjuvant therapy and intensified follow-up after surgery. Independent external validations may still be required.

## Background

Colorectal cancer (CRC) is a leading contributor to cancer mortality worldwide [1, 2]. Surgical treatment is the mainstay for elimination of CRC and continuity of life [3, 4]. However, patients with a high risk of postoperative progression of CRC require additional interventions and informed decision-making with the help of physicians [3–5]. Among the vast spectrum of clinicopathological information [3, 6], the American Joint Committee on Cancer (AJCC) stages of CRC are fundamental for choosing optimal clinical interventions, and their use remains at the forefront of predicting and treating CRC [7]. Unfortunately, many observations are not consistent with the assumed relationship between advanced anatomical stages and reduced survival probabilities. For instance, disease recurs in 25 % of patients with early CRC who are node-negative following curative resection [8]. Patients with stage II CRCs with low-risk features more frequently encounter adverse events than those with high-risk features [9]. Postoperative adjuvant therapies for patients with stage II CRC with fewer than 12 recovered nodes or other risk factors have not gained a clear survival benefit as expected [10–12]; however, a substantial improvement in survival has been achieved for patients with stage III CRC [11, 12]. Therapeutic effects only partially explain the conspicuous survival inhomogeneity within stage III CRC although stage migrations due to inadequate pathologic assessment may also play a role [13, 14]. Metastatic CRC after curative hepatic resection has a 5-year overall survival (OS) of 47.7 to 57.6 % [15, 16], while the OS of most patients with unresectable metastatic CRC is extremely poor [17]. Survival of CRC remains poor for multiple reasons that are not limited to tumor-related factors. Despite the increased complexity among several modifications of the AJCC cancer staging manuals [14], the AJCC stages have intrinsic defects as an anatomy-dependent rather than multidiscipline-integrated metric [18]. Moreover, the AJCC stages force categorization of tumor dissemination in a stepwise fashion, causing additional loss of predictive accuracy [18, 19]. A consequential issue has thus emerged: both the 5-year cancer-specific survival (CSS) and OS are heterogeneous among patients with the same stage of CRC [14].

Many useful factors are not sufficiently utilized in clinical prognostication. The plasma carcinoembryonic antigen (CEA) concentration is strongly predictive [15, 19] and plays roles in staging other than indicating recurrence. Patient-specific factors such as sex, ethnicity, and marital status are also associated with survival [1, 2, 20, 21], representing untapped information that may be useful for individualized therapies and outcomes. Many other parameters included in routine pathologic reports are also apparent survival determinants, including tumor location, size, histology, grade, differentiation, lymph node count (LNC), lymph node ratio (LNR), and surgical extent [6]. All of these elements are inseparable qualities of a “successful cancer career,” of which more detailed evaluations are still required, however.

We anticipate that the combined performance of the above-mentioned factors is superior to that of the AJCC stages and may serve as a more precise and reproducible tool for individualized survival estimations. We have herein incorporated clinically important variables with data from the Surveillance, Epidemiology, and End Results (SEER) database to develop validated prognostic nomograms for predicting CSS and OS of patients with surgically treated CRC without neoadjuvant therapies.

## Methods

### Patient eligibility and variables

The SEER program is a national database and primary source of cancer statistics that is currently maintained by the National Cancer Institute [22]. The data of patients with CRC diagnosed from 2004 to 2010 were retrieved from the SEER research database using the SEER*Stat program (v 8.2.1) [22]. In total, 265,030 records were retrieved. Any surgically treated, pathologically proven, staged colorectal adenocarcinomas were considered. Only patients who met the following criteria were included in the formal analysis: (1) known preoperative CEA status, (2) no history of malignancy, (3) microscopically proven stage I to IV primary adenocarcinoma (including signet ring cell carcinoma), (3) no adjuvant therapy before surgery, (4) cancer-directed surgery of primary tumors with sufficient information to specify the T/N/M stage and LNC/LNR, (5) active follow-up with complete date and known outcome, and (6) adequate/consistent information to specify the primary tumor site, size, and other variables. Patients aged <18 or >99 years and those with multiple primary cancers were excluded. Patients were also excluded if their T/N did not meet pathological staging criteria (not pT/N). These patients were small in number, but they might have introduced bias to the survival analysis; thus, they were excluded.

The following variables were assessed: sex, age, race, marital status, year of diagnosis, primary tumor location, size, histology, grade, TNM stage, LNC, cause-specific death, and vital status. Cancer stages reported using the 6th AJCC/TNM stages (AJCC6) were converted based on the 7th edition (AJCC7). The LNR was calculated by dividing metastatic node number by the LNC.

**Fig. 1:**
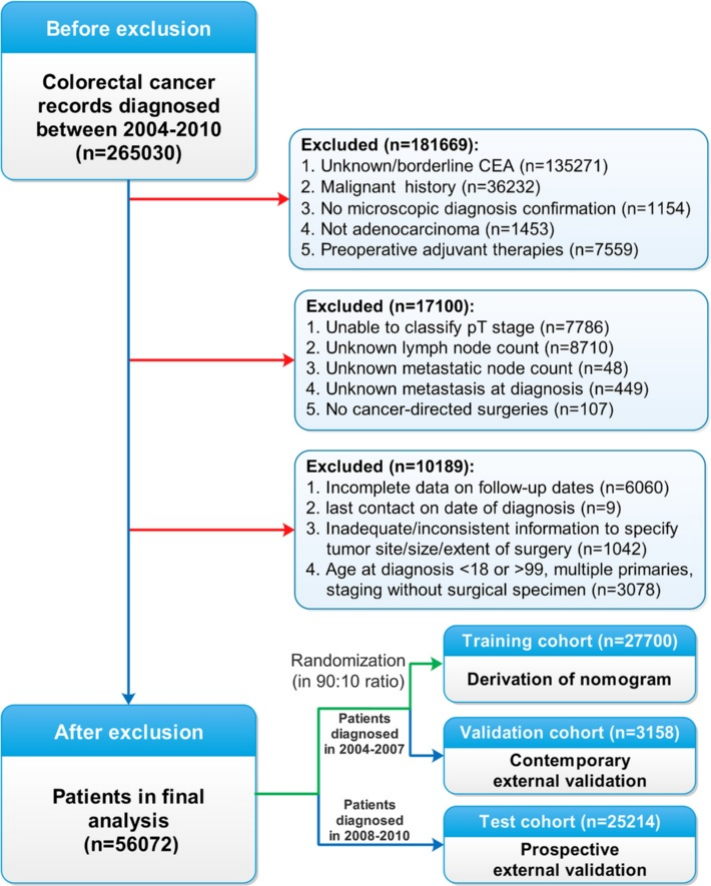
Flow diagram of patient selection and study development

After patient exclusion based on the above-mentioned criteria, 56,072 eligible patients were identified. Patients diagnosed from 2004 to 2007 were randomized into a training cohort and a contemporary validation cohort (ratio, 90:10). The remaining patients were diagnosed from 2008 to 2010 and were thus assigned to a prospective test cohort (Fig. 1).

### Statistical methods

Discontinuous variables were categorized before modeling based on clinical reasoning and significance. Linear assumptions of continuous variables (age, LNC, and LNR) were relaxed with restricted cubic spline functions to determine the optimal number of knots by maximizing goodness of fit using the log-likelihood and minimizing information loss using the Akaike information criterion (AIC) [23]. Multivariate models for nomograms were built by incorporating significant variables from univariate Cox proportional hazard regressions in a backward stepwise manner based on the AIC. Model performance was appraised using the concordance index (c-index) and internally testified by 200-sample bootstrap validation and calibration. External validation was performed by applying nomograms to the contemporary validation cohort and prospective test cohort separately, followed by evaluation of similar statistics in the new data sets. Different c-indexes were compared using the *compareC* [24] package. Next, patients in all cohorts were given an aggregated score using standard points derived from the nomograms. Time-dependent receiver-operating characteristic (ROC) curve analysis was performed with the *timeROC* [25] package to evaluate the performance of the nomograms with the accumulated scores as a continuous predictive variable. The nomograms were compared with the AJCC6/7 stages by risk classification and stratification using Kaplan–Meier survival curves and statistically clarified by quantifying the cumulative 5-year survival and hazard ratios for each stratum. Briefly, risk classification was achieved by ranking the accumulated nomogram scores by deciles to derive 10 risk groups (Nomo stages) with patients in the training cohort. For risk stratification, the patients were divided by score tertiles for each AJCC7 substage to generate three prognostic strata: low-, median-, and high-risk. The two external cohorts were likewise classified and stratified according to thresholds defined by the training cohort. Next, mosaic plots were drawn to demonstrate the AJCC7 stage distributions in contrast with the Nomo stages. After addressing the accuracy of the nomograms, decision curve analysis (DCA) [26] was performed to finalize the ranges of threshold probabilities within which the nomograms were clinically valuable. The patients were randomly allocated using the PASW 18.0 program (SPSS Inc., Chicago, IL); the other analyses were processed with the R program (v 3.2.3) using *rms* [23] and the above-mentioned packages. Only a two-tailed *P* value of <0.05 was considered statistically significant. This study followed the TRIPOD statement [27] and adhered to the Declaration of Helsinki for medical research involving human subjects [28].

## Results

### Baseline characteristics

The characteristics of the patients in the derivation and validation cohorts are shown in Table 1.

**Table 1:** Characteristics of patients with colorectal cancer

Variables	Training cohort		Validation cohort		Test cohort	
	(*n* = 27700)		(*n* = 3158)		(*n* = 25214)	
Sex, n, %						
Female	14077	50.8	1605	50.8	12702	50.4
Male	13623	49.2	1553	49.2	12512	49.6
Age, year, median, range	67	18–99	67	18–99	67	18–99
Race, n, %						
White	21722	78.4	2428	76.9	19710	78.2
Black	3422	12.4	412	13.0	3106	12.3
Yellow (Chinese, Korean and Japanese)	1229	4.4	169	5.4	1075	4.3
Other	1327	4.8	149	4.7	1323	5.2
Marital status at diagnosis, n, %						
Married (including separated)	15900	57.4	1812	57.3	14166	56.2
Divorced	2378	8.6	266	8.4	2297	9.1
Single (never married)	3519	12.7	406	12.9	3553	14.1
Widowed	5185	18.7	601	19.1	4363	17.3
Unknown	718	2.6	73	2.3	835	3.3
CEA status, n, %						
Negative	15550	56.1	1803	57.1	14824	58.8
Positive	12150	43.9	1355	42.9	10390	41.2
Tumor site, n, %						
Proximal colon (cecum to splenic flexure)	14341	51.7	1621	51.3	13790	54.7
Distal colon (descending to sigmoid colon)	8015	29.0	952	30.2	7441	29.5
Overlapping lesion of colon	284	1.0	24	0.8	275	1.1
Rectum (including rectosigmoid junction)	5060	18.3	561	17.7	3708	14.7
Tumor size, n, %						
≤ 5 cm	16861	60.9	1966	62.3	15178	60.2
> 5 cm	9120	32.9	998	31.6	8557	33.9
Unknown	1719	6.2	194	6.1	1479	5.9
Extent of surgery, n, %						
Local/segmental resection	12879	46.5	1505	47.7	11464	45.5
Subtotal/hemisection	13991	50.5	1549	49.0	13137	52.1
Total resection	830	3.0	104	3.3	613	2.4
Histology, n, %						
Adenocarcinoma	27375	98.8	3116	98.7	24975	99.1
Signet ring cell carcinoma	325	1.2	42	1.3	239	0.9
Tumor grade, n, %						
Well to Moderately differentiated (G1 + G2)	21137	76.3	2435	77.1	19450	77.2
Poorly to Undifferentiated (G3 + G4)	5931	21.4	646	20.5	5202	20.6
Unknown	632	2.3	77	2.4	562	2.2
pT stage, n, %						
pT1	2381	8.6	272	8.6	2662	10.6
pT2	3987	14.4	447	14.2	3844	15.2
pT3	17094	61.7	1980	62.7	14887	59.0
pT4a	2244	8.1	256	8.1	2242	8.9
pT4b	1994	7.2	203	6.4	1579	6.3
pN stage, n, %						
N0	14069	50.8	1615	51.1	13378	53.1
N1a	3445	12.4	402	12.7	3005	11.9
N1b	3960	14.3	456	14.4	3378	13.4
N2a	3055	11.0	356	11.2	2611	10.4
N2b	3171	11.5	329	10.4	2842	11.2
Lymph node count, mean, sd	15.7	9.6	15.8	9.6	18.4	9.6
Lymph node ratio, mean, IQR	0.16	0–0.24	0.16	0–0.22	0.13	0–0.18
Metastasis, n, %						
M0	22512	81.3	2587	81.9	21112	83.7
M1	5188	18.7	571	18.1	4102	16.3
Follow-up	63	1–107	64	1–107	34	1–59
Number of events	9341	13359	1055	1496	5659	7689
1-year cumulative survival	87.9	84.1	88.6	84.7	89.8	86.5
3-year cumulative survival	73.8	67.1	75.1	68.7	77.3	70.9
5-year cumulative survival ^a^	66.6	57.2	67.7	58.6	70.8	60.6

^a^Survival probabilities of the test cohort at 5 years were approximated at 59 months

*CEA* carcinoembryonic antigen, *sd* standard deviation, *IQR* interquartile range, *CSS* cancer-specific survival, *OS* overall survival

### Cox regression of training cohort

**Fig. 2:**
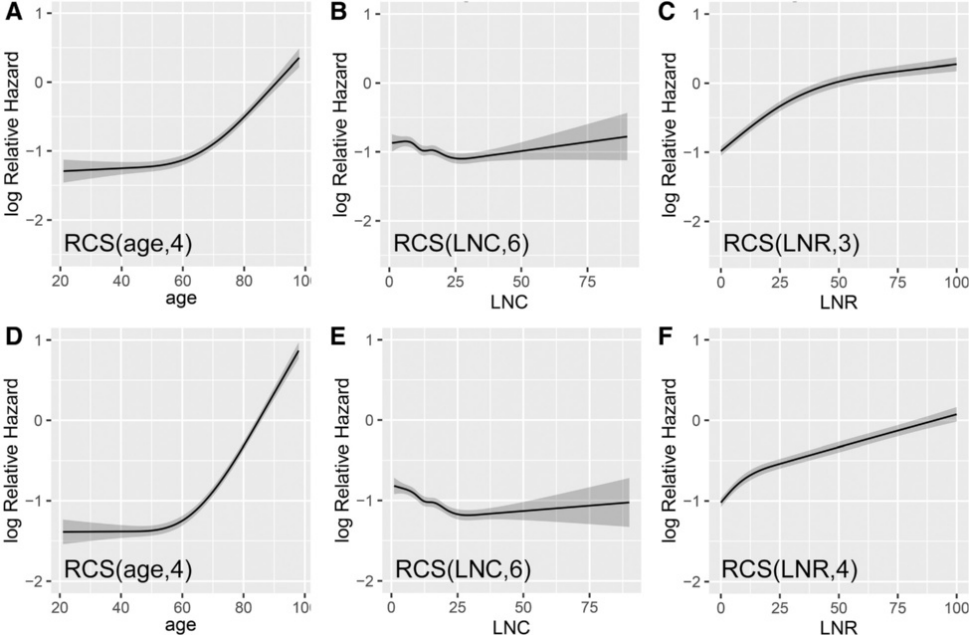
Adjusted relative hazards with continuous variables. **a**–**c ** The optimized number of knots applied in the multivariate analysis of CSS was 4, 6, and 3 for age, LNC, and LNR, respectively. **d**–**f** These numbers of knots were 4, 6, and 4 for the same three variables in the analysis of OS. RCS, restricted cubic spline function; CSS, cancer-specific survival; OS, overall survival; LNC, lymph node count; LNR, lymph node ratio

No continuous variables (age, LNC, or LNR) had linear effects on either CSS or OS (Fig. 2). All variables assessed in the univariate analysis (Table 2) remained significant in the multivariate Cox regressions except tumor size (Table 3).

**Table 2:** Univariate cox regression analysis of training cohort

Variables	Cancer-specific survival			Overall survival		
	HR	95 % CI	*P*	HR	95 % CI	*P*
Sex						
Female	ref			ref		
Male	1.060	1.018–1.104	0.0049	1.055	1.019–1.091	0.0021
Race						
White	ref			ref		
Black	1.278	1.206–1.354	<0.0001	1.157	1.101–1.216	<0.0001
Yellow (Chinese, Korean and Japanese)	0.762	0.682–0.850	<0.0001	0.701	0.638–0.770	<0.0001
Other	0.974	0.884–1.072	0.5850	0.870	0.800–0.946	0.0011
Marital status at diagnosis						
Married (including separated)	ref			ref		
Divorced	1.171	1.088–1.259	<0.0001	1.175	1.103–1.251	<0.0001
Single (never married)	1.332	1.255–1.414	<0.0001	1.316	1.250–1.386	<0.0001
Widowed	1.376	1.305–1.450	<0.0001	1.746	1.675–1.821	<0.0001
Unknown	1.045	0.914–1.196	0.5175	1.246	1.120–1.387	0.0001
CEA status						
Negative	ref			ref		
Positive	3.174	3.042–3.313	<0.0001	2.449	2.366–2.535	<0.0001
Tumor site						
Proximal colon	ref			ref		
Distal colon	0.892	0.850–0.935	<0.0001	0.835	0.803–0.869	<0.0001
Overlapping lesion of colon	1.382	1.153–1.657	0.0005	1.294	1.108–1.510	0.0011
Rectum	0.880	0.833–0.931	<0.0001	0.825	0.787–0.865	<0.0001
Tumor size						
≤ 5 cm	ref			ref		
> 5 cm	1.429	1.370–1.491	<0.0001	1.316	1.270–1.363	<0.0001
Unknown	0.821	0.746–0.904	0.0001	0.806	0.745–0.873	<0.0001
Extent of surgery						
Local/segmental resection	ref			ref		
Subtotal/hemisection	1.161	1.114–1.211	<0.0001	1.181	1.141–1.223	<0.0001
Total resection	1.527	1.373–1.699	<0.0001	1.388	1.264–1.525	<0.0001
Histology						
Adenocarcinoma	ref			ref		
Signet ring cell carcinoma	2.648	2.304–3.044	<0.0001	2.180	1.916–2.480	<0.0001
Tumor grade						
G1/G2	ref			ref		
G3/G4	1.903	1.820–1.989	<0.0001	1.614	1.553–1.678	<0.0001
Unknown	1.007	0.871–1.164	0.9283	0.942	0.834–1.064	0.3372
pT stage						
pT1	ref			ref		
pT2	1.854	1.556–2.209	<0.0001	1.662	1.495–1.848	<0.0001
pT3	6.161	5.285–7.182	<0.0001	3.150	2.871–1.848	<0.0001
pT4a	13.422	11.43–15.760	<0.0001	5.775	5.21–6.400	<0.0001
pT4b	17.429	14.844–20.464	<0.0001	7.324	6.607–8.120	<0.0001
Metastasis						
M0	ref			ref		
M1	7.733	7.414–8.066	<0.0001	4.953	4.773–5.139	<0.0001

*HR* hazard ratio, *95 % CI* 95 % confident interval, *ref* reference category, *CEA* carcinoembryonic antigen

**Table 3:** Multivariate cox regression analysis of training cohort

	Cancer-specific survival			Overall survival		
Covariates	HR	95 % CI	*P*	HR	95 % CI	*P*
Sex (Male vs. Female)	1.142	1.094–1.193	<0.0001	1.246	1.202–1.293	<0.0001
Race (ref, White)						
Black	1.199	1.130–1.273	<0.0001	1.191	1.132–1.254	<0.0001
Yellow (Chinese, Korean, Japanese)	0.784	0.702–0.875	<0.0001	0.713	0.649–0.784	<0.0001
Other	1.030	0.935–1.135	0.5449	0.996	0.915–1.083	0.9203
Marital status at diagnosis (ref, Married)						
Divorced	1.175	1.092–1.265	<0.0001	1.214	1.139–1.293	<0.0001
Single (never married)	1.235	1.161–1.313	<0.0001	1.277	1.211–1.346	<0.0001
Widowed	1.189	1.118–1.264	<0.0001	1.224	1.165–1.285	<0.0001
Unknown	1.014	0.886–1.161	0.8424	1.145	1.029–1.275	0.0131
CEA status (Positive vs. negative)	1.589	1.517–1.664	<0.0001	1.486	1.431–1.543	<0.0001
Extent of surgery (ref, Loc/seg resection)						
Subtotal/hemisection	1.067	1.012–1.125	0.0156	1.040	0.995–1.087	0.0824
Total resection	1.269	1.139–1.414	<0.0001	1.180	1.073–1.297	0.0007
Tumor site (ref, Proximal colon)						
Distal colon	0.910	0.860–0.962	<0.0001	0.918	0.876–0.962	0.0004
Overlapping lesion of colon	1.090	0.909–1.307	0.3545	1.111	0.952–1.298	0.1824
Rectum	1.002	0.935–1.074	0.9516	0.981	0.926–1.040	0.5211
Tumor size (ref, ≤ 5 cm)						
>5 cm	1.029	0.984–1.076	0.2050	1.026	0.989–1.066	0.1741
Unknown	1.141	1.035–1.258	0.0078	1.075	0.991–1.166	0.0810
Histology (ref, Adenocarcinoma)						
Signet ring cell carcinoma	1.409	1.220–1.626	<0.0001	1.380	1.209–1.575	<0.0001
Tumor grade (ref, G1/G2)						
G3/G4	1.278	1.219–1.339	<0.0001	1.184	1.137–1.233	<0.0001
Unknown	1.143	0.987–1.323	0.0736	1.049	0.927–1.186	0.4501
pT stage (ref, pT1)						
pT2	1.567	1.312–1.872	<0.0001	1.398	1.254–1.558	<0.0001
pT3	2.949	2.516–3.457	<0.0001	1.796	1.628–1.981	<0.0001
pT4a	4.429	3.746–5.237	<0.0001	2.508	2.247–2.799	<0.0001
pT4b	4.760	4.021–5.634	<0.0001	2.746	2.456–3.069	<0.0001
Metastasis (M1 vs. M0)	4.075	3.876–4.284	<0.0001	3.357	3.213–3.508	<0.0001

*HR* hazard ratio, *95 % CI* 95 % confident interval, *ref* reference category, *Loc/seg resection* Local/segmental resection, *CEA* carcinoembryonic antigen

### Nomograms for CSS and OS

**Fig. 3:**
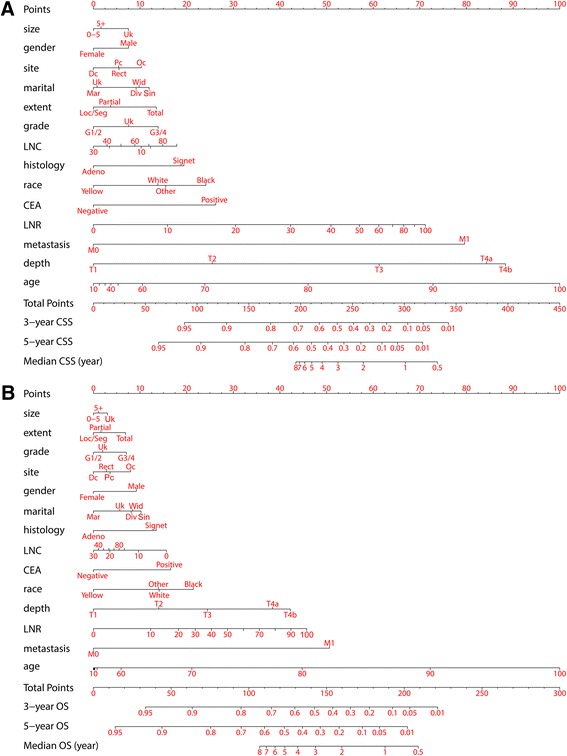
Nomograms for (**a**) CSS and (**b**) OS. Generally, each individual involved covariate was assessed for the patient and given a point on the basis of the nomograms. Aggregated points were obtained by summing the given points of all involved variables. The aggregated points corresponded to particular survival probabilities and median survival years that could be indicated by the nomograms. A higher total number of points frequently indicated a higher possibility of adverse outcomes (CSS or OS) and therefore a lower expected survival. CSS, cancer-specific survival; OS, overall survival

As selected by the AIC, all tested covariates were employed in the nomograms. The c-indexes were 0.816 (95 % CI 0.810–0.822) and 0.777 (95 % CI 0.772–0.782) for the CSS (Fig. 3a) and OS (Fig. 3b) predictive nomograms, respectively. Details of the nomograms’ labels and points were shown in Table 4 and Table 5.

**Table 4:** Points for categorical variables in nomograms

		Points	
Variables	Labels for tick marks	CSS	OS
Sex			
Female	Female	0	0
Male	Male	7.5	9.2
Race			
White	White	13.8	14.1
Black	Black	24.1	21.4
Yellow (Chinese, Korean and Japanese)	Yellow	0	0
Other	Other	15.5	13.9
Marital status at diagnosis			
Married (including separated)	Mar	0	0
Divorced	Div	9.2	8.1
Single (never married)	Sin	11.9	10.2
Widowed	Wid	9.8	8.4
Unknown	Uk	0.8	5.7
CEA status			
Negative	Negative	0	0
Positive	Positive	26.2	16.6
Tumor site			
Proximal colon (cecum to splenic flexure)	Pc	5.4	3.6
Distal colon (descending to sigmoid colon)	Dc	0	0
Overlapping lesion of colon	Oc	10.3	8.0
Rectum (including rectosigmoid junction)	Rect	5.5	2.8
Tumor size			
≤5 cm	0–5	0	0
>5 cm	5+	1.6	1.1
Unknown	Uk	7.5	3.0
Extent of surgery			
Local/segmental resection	Loc/Seg	0	0
Subtotal/hemisection	Partial	3.7	1.6
Total resection	Total	13.5	6.9
Histology			
Adenocarcinoma	Adeno	0	0
Signet ring cell carcinoma	Signet	19.4	13.5
Tumor grade			
Well to Moderately differentiated (G1 + G2)	G1/2	0	0
Poorly to Undifferentiated (G3 + G4)	G3/4	13.9	7.1
Unknown	Uk	7.6	2.0
pT stage			
pT1	T1	0	0
pT2	T2	25.5	14.0
pT3	T3	61.3	24.5
pT4a	T4a	84.3	38.4
pT4b	T4b	88.4	42.2
Metastasis			
M0	M0	0	0
M1	M1	79.6	50.6

*CSS* cancer-specific survival, *OS* overall survival, *CEA* carcinoembryonic antigen

**Table 5:** Points for continuous variables in nomograms

Age at diagnosis			Lymph node count, n			Lymph node ratio		
Values, no.	Pts for CSS	Pts for OS	Values, no.	Pts for CSS	Pts for OS	Values, %	Pts for CSS	Pts for OS
10	0.0	0.0	0	12.3	15.7	0	0.0	0.0
20	1.2	0.1	10	10.2	9.7	10	15.9	12.3
30	2.5	0.2	20	3.4	3.5	20	30.5	18.2
40	3.7	0.3	30	0.0	0.0	30	42.3	21.8
50	5.3	0.8	40	2.9	1.1	40	50.9	25.3
60	10.5	5.9	50	5.9	2.2	50	57.0	28.8
70	23.9	21.1	60	8.9	3.3	60	61.2	32.2
80	46.0	44.8	70	11.9	4.4	70	64.2	35.6
90	72.8	72.2	80	14.9	5.5	80	66.5	39.0
100	100.0	100.0	90	17.9	6.6	90	68.9	42.4
						100	71.2	45.7

*Pts* points, *CSS* cancer-specific survival, *OS* overall survival

### Internal validation

The bootstrap-corrected c-indexes (CSS, 0.8157; OS, 0.7768) were close to those of the nomograms. Both models exhibited good validation.

### Nomogram calibration

**Fig. 4:**
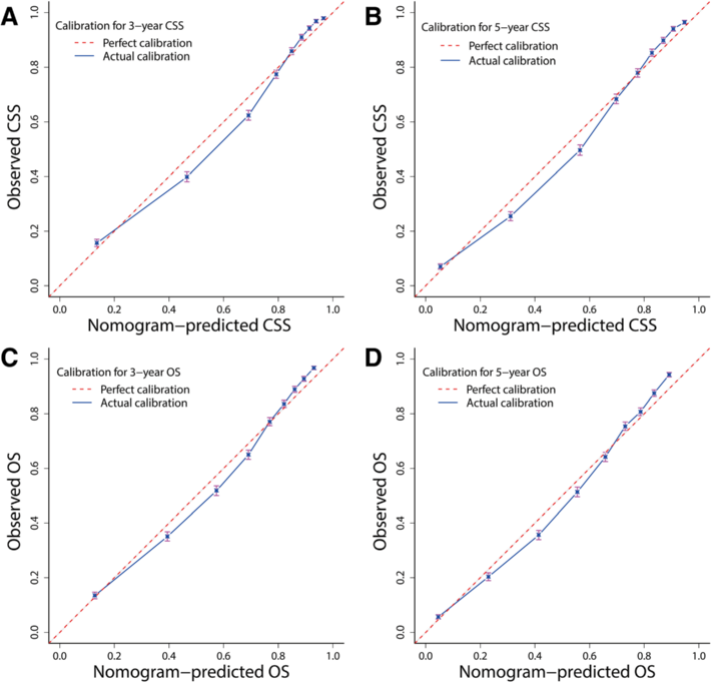
Bootstrap calibrations of nomograms. The predicted 3- and 5-year (**a**, **b**) CSS and (**c**, **d**) OS were well correlated with the actual survival probabilities

As shown in the calibration plots, the nomogram-predicted 3- and 5-year CSS and OS were well correlated with the corresponding Kaplan–Meier estimates (Fig. 4), suggesting appreciable reliability of the nomograms.

### External validation

**Fig. 5:**
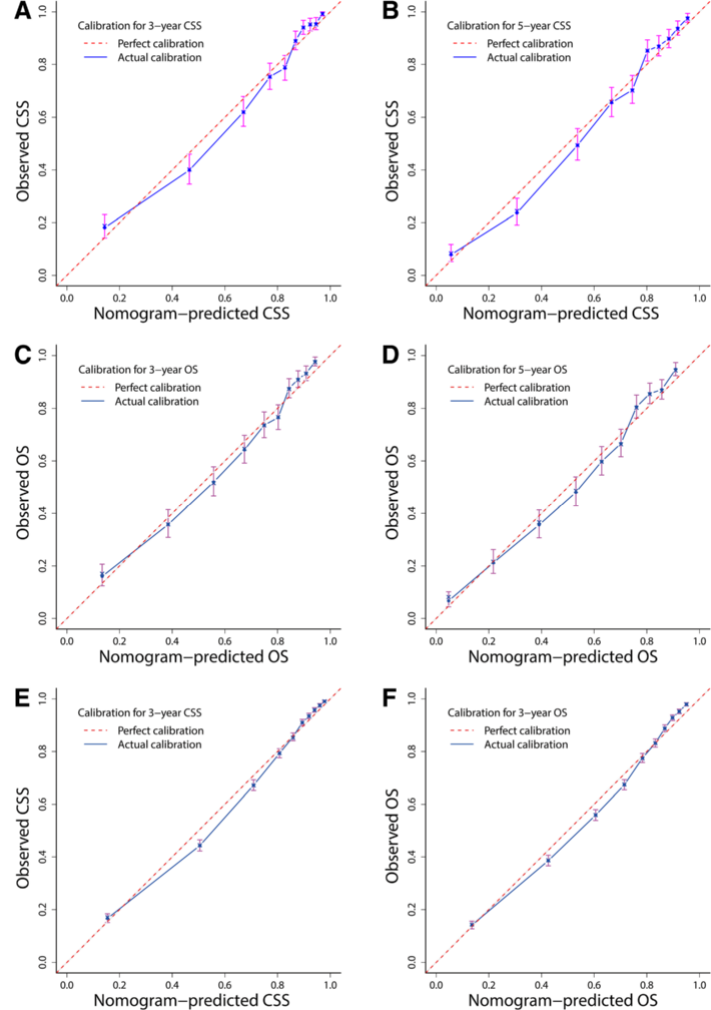
Bootstrap calibration of nomograms in the external cohorts. The nomograms were externally calibrated in the validation cohort by predicting the 3- and 5-year (**a**, **b**) CSS and (**c**, **d**) OS. The nomograms were also calibrated in the test cohort for the 3-year (**e**) CSS and (**f**) OS. All results showed good validation. CSS, cancer-specific survival; OS, overall survival

The c-indexes of the nomograms for prediction of CSS and OS were 0.809 (95 % CI 0.791–0.827) and 0.773 (95 % CI 0.757–0.789) in the validation cohort, while the optimism-corrected c-indexes were 0.804 and 0.768, respectively. In the test cohort, the c-indexes were 0.839 (95 % CI 0.830–0.846) and 0.802 (95 % CI 0.796–0.808) with corrected estimates of 0.838 and 0.801 for CSS and OS prediction, respectively. The external calibration plots also showed good validation (Fig. 5).

### Time-dependent ROC curve analysis

The areas under the ROC curve (AUCs) for predicting CSS ranged from 83.0 to 87.8 % in the three cohorts from 1 to 7 years. The AUCs for predicting OS varied from 80.6 to 84.6 % during the same years (Table 6). The nomograms exhibited considerable efficiency to discriminate outcomes.

**Table 6:** Time-dependent receiver-operating characteristic curves analysis

	Cancer-specific survival				Overall survival			
	AUC, %				AUC, %			
Study cohort	1 year	3 years	5 years	7 years	1 year	3 years	5 years	7 years
Training cohort	85.2	87.6	87.5	86.6	82.1	84.1	84.2	83.6
Validation cohort	83.0	87.1	86.7	85.5	80.6	83.7	83.7	82.7
Test cohort	86.0	87.8	/	/	83.1	84.6	/	/

*AUC* area under the time-dependent receiver-operating characteristic curves

### Comparison of nomograms with AJCC stages

First, when compared with the AJCC6/7 stages, the nomograms yielded the largest log-likelihoods and c-indexes along with the smallest AIC values for CSS and OS prediction in all cohorts (Table 7). These results imply that the nomograms were more robust than the AJCC stages.

**Table 7:** Comparison of nomogram with AJCC staging system

	Nomogram score	7^th^ AJCC stage	6^th^ AJCC stage	*P*
Training cohort, CSS				
AIC	172262	174703	174949	/
Log-likelihood	−86130	−87344	−87468	All <0.0001
C-index (95 % CI)	0.816 (0.810–0.822)	0.777 (0.771–0.783)	0.774 (0.768–0.780)	All <0.0001
Training cohort, OS				
AIC	250348	255973	256182	/
Log-likelihood	−125173	−127979	−128085	All <0.0001
C-index (95 % CI)	0.777 (0.772–0.782)	0.698 (0.693–0.0.703)	0.696 (0.691–0.701)	All <0.0001
Validation cohort, CSS				
AIC	14983	15261	15272	/
Log-likelihood^a^	−7490	−7623	−7630	All <0.0001
C-index^b^ (95 % CI)	0.809 (0.791–0.827)	0.770 (0.752–0.788)	0.768 (0.750–0.786)	All <0.0001
Validation cohort, OS				
AIC	21611	22235	22244	/
Log-likelihood^c^	−10805	−11110	−11116	All <0.0001
C-index^d^ (95 % CI)	0.773 (0.757–0.789)	0.699 (0.683–0.715)	0.697 (0.681–0.713)	All <0.0001
Test cohort, CSS				
AIC	102103	104057	105039	/
Log-likelihood	−51050	−52021	−52519	All <0.0001
C-index (95 % CI)	0.838 (0.830–0.846)	0.794 (0.786–0.802)	0.786 (0.778–0.794)	All <0.0001
Test cohort, OS				
AIC	141606	145343	146456	/
Log-likelihood	−70802	−72664	−73227	All <0.0001
C-index (95 % CI)	0.802 (0.796–0.808)	0.723 (0.717–0.729)	0.715 (0.709–0.721)	All <0.0001

*AJCC* American joint committee on cancer, *AIC* akaike information criterion, *C-index* concordance index, *95 % CI* 95 % confident interval

^a^The *P* value comparing 6^th^ and 7^th^ AJCC stage was 0.0003

^b^The *P* value comparing 6^th^ and 7^th^ AJCC stage was 0.5187

^c^The *P* value comparing 6^th^ and 7^th^ AJCC stage was 0.0006

^d^The *P* value comparing 6^th^ and 7^th^ AJCC stage was 0.3709

**Fig. 6:**
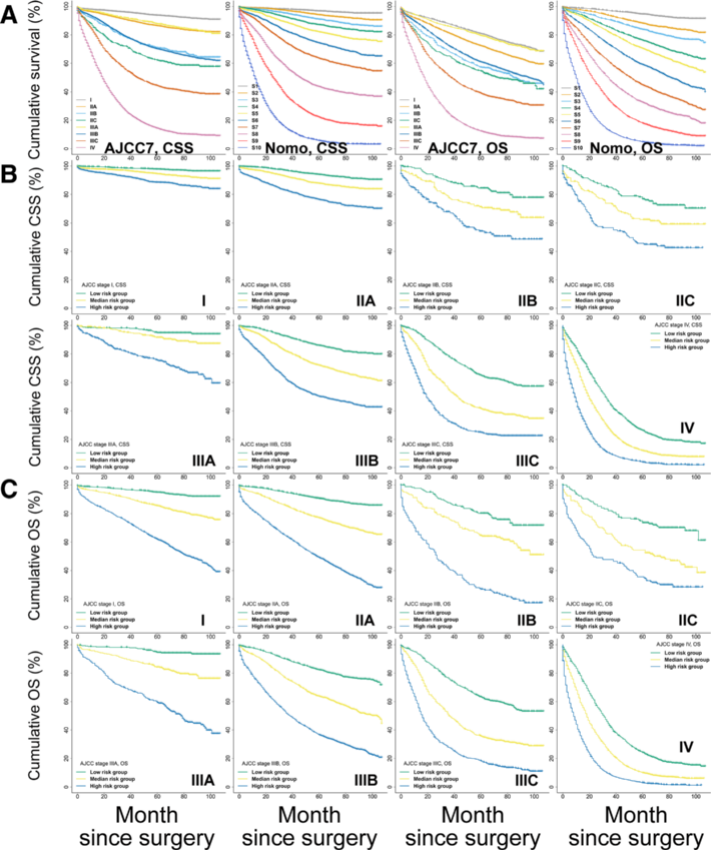
Kaplan–Meier curve analysis of risk classification and stratification in the training cohort. **a** Risk classification of CSS and OS using the 7th edition of the AJCC stages (AJCC7) and Nomo stages (all log-rank *P* values for trend <0.0001). **b** Risk stratification of CSS for each AJCC7 substage. **c** Risk stratification of OS for each AJCC7 substage (all log-rank *P* values for trend <0.0001, all log-rank *P* values for pairwise comparisons <0.05). CSS, cancer-specific survival; OS, overall survival; AJCC, American Joint Committee on Cancer

**Fig. 7:**
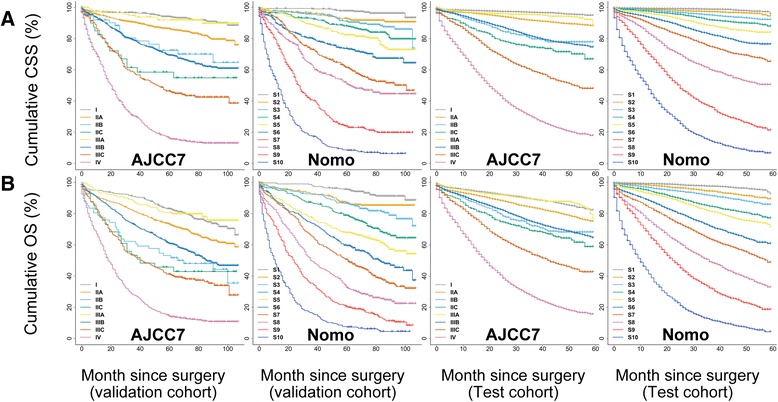
Kaplan–Meier curve analysis for risk classification of (**a**) CSS and (**b**) OS in the validation and test cohorts. All log-rank *P* values for trend <0.0001. CSS, cancer-specific survival; OS, overall survival

**Fig. 8:**
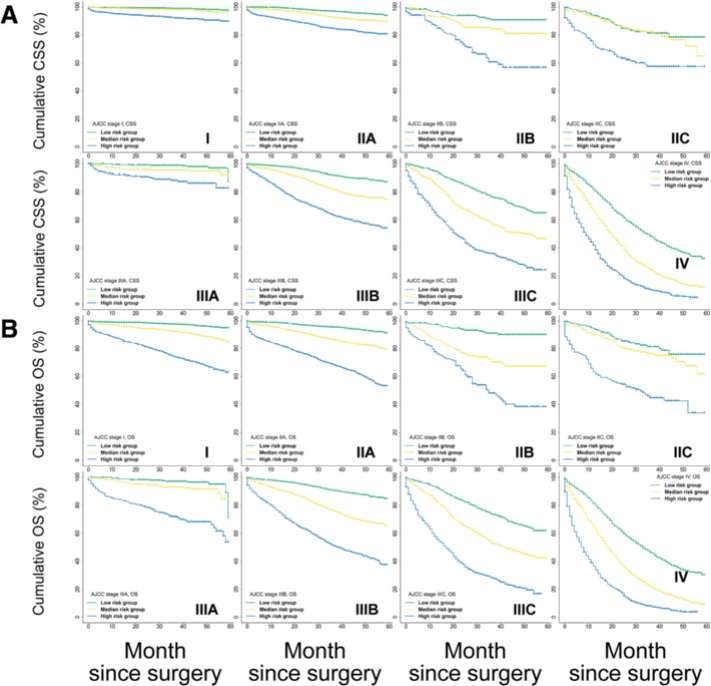
Kaplan–Meier curve analysis for risk stratification of (**a**) CSS and (**b**) OS within each AJCC substage using the test cohort. All log-rank *P* values for trend <0.0001. CSS, cancer-specific survival; OS, overall survival

Second, as depicted by the Kaplan–Meier curves, the ability of the AJCC7 stages to discriminate CSS and OS was mediocre in both the training (Fig. 6a) and external cohorts (Fig. 7). However, the Nomo stages performed consistently much better in all cohorts (Figs. 6a and 7). Further analysis in the training (Fig. 6b and c) and test cohorts (Fig. 8) showed that the nomograms were also able to stratify each AJCC7 stage into three significant prognostic groups with low, medium, and high risks of CSS and OS, respectively. Additional elaborations on the 5-year cumulative survival (Table 8) and hazard ratios (Table 9) of the Nomo stages as well as the stratified risk groups (Table 10) confirmed robust utility of nomograms in both risk classification and stratification.

**Table 8:** Cumulative survival for Nomo stages in derivation and external validation cohorts

	Training cohort				Validation cohort				Test cohort			
	(Cumulative survival, 60 months, %)				(Cumulative survival, 60 months, %)				(Cumulative survival, 59 months, %)			
Nomo stage	CSS	95 % CI	OS	95 % CI	CSS	95 % CI	OS	95 % CI	CSS	95 % CI	OS	95 % CI
Nomo 1	96.8	96.1–97.4	94.6	93.7–95.4	97.7	96.1–99.4	95.5	93.2–97.8	94.3	88.7–99.8	90.8	85.5–96.1
Nomo 2	94.0	93.1–95.0	88.1	86.9–89.3	92.9	90.0–95.8	87.1	83.4–90.9	94.0	91.5–96.6	89.0	86.5–91.4
Nomo 3	90.9	89.8–92.0	83.7	82.4–85.1	91.2	88.0–94.4	86.6	82.8–90.3	92.1	90.5–93.6	85.5	83.4–87.7
Nomo 4	87.1	85.8–88.4	76.1	74.5–77.7	88.1	84.4–91.7	78.9	74.4–83.4	87.4	84.5–90.3	76.5	73.5–79.5
Nomo 5	82.0	80.4–83.5	70.3	68.6–72.0	82.3	77.9–86.7	67.8	62.6–73.0	83.7	81.8–85.7	69.1	62.1–76.1
Nomo 6	74.2	72.4–75.9	59.0	57.2–60.9	73.6	68.5–78.7	59.4	54.0–64.8	76.1	73.9–78.4	60.1	56.9–63.3
Nomo 7	63.3	61.4–65.2	44.8	42.9–46.7	61.3	55.6–67.0	50.6	45.1–56.1	64.4	60.3–68.6	47.4	43.3–51.5
Nomo 8	43.6	41.6–45.6	32.6	30.9–34.4	50.8	44.8–56.8	32.7	27.5–37.9	49.9	46.9–53.0	31.8	27.9–35.7
Nomo 9	22.1	20.4–23.7	18.0	16.5–19.4	23.9	18.8–29.0	20.2	15.8–24.7	20.2	16.0–24.4	17.8	15.0–20.6
Nomo 10	5.7	4.7–6.6	4.8	4.0–5.6	8.4	5.2–11.6	7.3	4.5–10.2	6.3	3.9–8.6	3.8	1.3–6.3
*P*_log-rank_ for trend	<0.0001		<0.0001		<0.0001		<0.0001		<0.0001		<0.0001	

*CSS* cancer-specific survival, *OS* overall survival, *Nomo* Nomo stages

**Table 9:** Relative hazard for Nomo stages in derivation and external validation cohorts

		Training cohort			Validation cohort			Test cohort		
Nomo stages	Cut-off Points	HR	95 % CI	*P*	HR	95 % CI	*P*	HR	95 % CI	*P*
Cancer-specific survival										
Nomo 1	≤82.0	ref			ref			ref		
Nomo 2	≤106.2	1.86	1.48–2.35	<0.0001	2.40	1.19–4.84	0.0147	1.92	1.37–2.70	0.0002
Nomo 3	≤122.9	2.86	2.30–3.55	<0.0001	3.33	1.70–6.51	0.0004	3.38	2.48–4.62	<0.0001
Nomo 4	≤138.2	3.91	3.17–4.82	<0.0001	4.83	2.54–9.22	<0.0001	5.13	3.81–6.91	<0.0001
Nomo 5	≤153.0	5.58	4.55–6.84	<0.0001	6.60	3.50–12.42	<0.0001	7.87	5.89–10.51	<0.0001
Nomo 6	≤170.7	8.47	6.95–10.33	<0.0001	10.41	5.60–19.32	<0.0001	12.17	9.19–16.13	<0.0001
Nomo 7	≤192.2	12.46	10.25–15.14	<0.0001	16.23	8.84–29.81	<0.0001	18.39	13.91–24.3	<0.0001
Nomo 8	≤225.6	22.40	18.49–27.13	<0.0001	21.11	11.53–38.67	<0.0001	32.19	24.47–42.34	<0.0001
Nomo 9	≤272.1	40.47	33.46–48.94	<0.0001	42.87	23.56–78.00	<0.0001	65.32	49.79–85.68	<0.0001
Nomo 10	272.1+	83.31	68.9–100.73	<0.0001	86.07	47.39–156.31	<0.0001	127.46	97.2–167.12	<0.0001
Overall survival										
Nomo 1	≤57.2	ref			ref			ref		
Nomo 2	≤70.0	2.16	1.83–2.56	<0.0001	1.96	1.19–3.22	0.0078	2.13	1.68–2.70	<0.0001
Nomo 3	≤80.8	3.04	2.59–3.58	<0.0001	2.63	1.64–4.21	0.0001	3.12	2.49–3.92	<0.0001
Nomo 4	≤90.7	4.76	4.08–5.56	<0.0001	4.28	2.73–6.71	<0.0001	5.55	4.48–6.87	<0.0001
Nomo 5	≤101.6	6.12	5.26–7.12	<0.0001	6.17	3.99–9.55	<0.0001	6.90	5.59–8.52	<0.0001
Nomo 6	≤114.2	9.01	7.76–10.45	<0.0001	8.41	5.47–12.92	<0.0001	11.11	9.06–13.63	<0.0001
Nomo 7	≤129.1	13.46	11.62–15.58	<0.0001	11.25	7.36–17.19	<0.0001	15.23	12.45–18.62	<0.0001
Nomo 8	≤147.1	18.68	16.15–21.61	<0.0001	16.81	11.04–25.61	<0.0001	24.23	19.85–29.57	<0.0001
Nomo 9	≤171.8	28.44	24.61–32.88	<0.0001	25.47	16.76–38.68	<0.0001	37.66	30.91–45.89	<0.0001
Nomo 10	171.8+	55.25	47.8–63.86	<0.0001	45.27	29.80–68.76	<0.0001	75.07	61.63–91.45	<0.0001

*HR* hazard ratio, *95 % CI* 95 % confident interval

**Table 10:** Risk stratifications for each AJCC substage in training and test cohorts

		Training cohort				Test cohort		
AJCC stages	Cut-off Points	Cumulative Survival, 60 months, %	HR	95 % CI	Pairwise *P*_log-rank_	HR	95 % CI	Pairwise *P*_log-rank_
Cancer-specific survival								
Stage I								
Low risk group (L)	≤70.0	97.7	ref		L v M < 0.0001	ref		L v M < 0.0205
Median risk group (M)	≤97.2	94.4	2.44	1.91–3.13	L v H < 0.0001	1.75	1.24–2.45	L v H < 0.0001
High risk group (H)	97.2+	88.4	4.73	3.66–6.12	M v H < 0.0001	6.12	4.27–8.77	M v H < 0.0001
Stage IIA								
Low risk group (L)	≤122.7	93.9	ref		L v M < 0.0001	ref		L v M < 0.0001
Median risk group (M)	≤149.4	87.7	1.90	1.64–2.19	L v H < 0.0001	2.11	1.4–2.58	L v H < 0.0001
High risk group (H)	149.4+	76.2	3.96	3.41–4.61	M v H < 0.0001	4.66	3.80–5.72	M v H < 0.0001
Stage IIB								
Low risk group (L)	≤151.2	84.4	ref		L v M = 0.0046	ref		L v M = 0.0220
Median risk group (M)	≤178.4	71.1	1.84	1.28–2.64	L v H < 0.0001	2.09	1.29–3.39	L v H < 0.0001
High risk group (H)	178.4+	54.1	3.30	2.22–4.90	M v H < 0.0012	5.27	3.16–8.81	M v H < 0.0001
Stage IIC								
Low risk group (L)	≤158.0	76.7	ref		L v M = 0.0121	ref		L v M = 0.6571
Median risk group (M)	≤181.8	62.3	1.64	1.16–2.31	L v H < 0.0001	1.12	0.72–1.74	L v H < 0.0001
High risk group (H)	181.8+	45.6	2.79	1.93–2.03	M v H < 0.0020	2.73	1.68–4.42	M v H < 0.0001
Stage IIIA								
Low risk group (L)	≤88.9	94.8	ref		L v M = 0.0116	ref		L v M < 0.0210
Median risk group (M)	≤120.2	91.6	2.04	1.39–2.99	L v H < 0.0001	2.51	1.36–4.63	L v H < 0.0001
High risk group (H)	120.2+	75.6	6.84	4.57–10.24	M v H < 0.0001	6.54	3.20–13.35	M v H = 0.0015
Stage IIIB								
Low risk group (L)	≤147.6	85.9	ref		L v M < 0.0001	ref		L v M < 0.0001
Median risk group (M)	≤180.3	72.1	2.09	1.89–2.32	L v H < 0.0001	2.35	2.04–2.70	L v H < 0.0001
High risk group (H)	180.3+	50.1	4.25	3.80–4.76	M v H < 0.0001	5.34	4.55–2.67	M v H < 0.0001
Stage IIIC								
Low risk group (L)	≤187.2	65.1	ref		L v M < 0.0001	ref		L v M < 0.0001
Median risk group (M)	≤218.0	41.6	1.99	1.74–2.27	L v H < 0.0001	2.01	1.72–2.36	L v H < 0.0001
High risk group (H)	218.0+	24.8	3.49	3.01–4.04	M v H < 0.0001	4.05	3.39–4.84	M v H < 0.0001
Stage IV								
Low risk group (L)	≤255.6	26.2	ref		L v M < 0.0001	ref		L v M < 0.0001
Median risk group (M)	≤292.1	12.0	1.56	1.46–1.67	L v H < 0.0001	1.85	1.70–2.01	L v H < 0.0001
High risk group (H)	292.1+	4.0	2.78	2.57–3.01	M v H < 0.0001	3.34	3.20–3.70	M v H < 0.0001
Overall survival								
Stage I								
Low risk group (L)	≤55.1	94.6	ref		L v M < 0.0001	ref		L v M < 0.0001
Median risk group (M)	≤79.9	84.5	2.94	2.58–3.35	L v H < 0.0001	3.10	2.58–3.72	L v H < 0.0001
High risk group (H)	79.9+	63.1	9.07	7.90–10.41	M v H < 0.0001	10.70	8.80–13.01	M v H < 0.0001
Stage IIA								
Low risk group (L)	≤75.9	90.6	ref		L v M < 0.0001	ref		L v M < 0.0001
Median risk group (M)	≤101.4	77.0	2.66	2.42–2.91	L v H < 0.0001	2.86	2.50–3.28	L v H < 0.0001
High risk group (H)	101.4+	49.1	7.38	6.68–8.15	M v H < 0.0001	7.80	6.77–8.97	M v H < 0.0001
Stage IIB								
Low risk group (L)	≤93.5	80.3	ref		L v M = 0.0007	ref		L v M < 0.0001
Median risk group (M)	≤120.4	64.7	1.86	1.40–2.45	L v H < 0.0001	3.86	2.60–5.72	L v H < 0.0001
High risk group (H)	120.4+	26.8	5.48	4.00–7.49	M v H < 0.0001	8.03	5.27–12.22	M v H < 0.0001
Stage IIC								
Low risk group (L)	≤96.9	73.6	ref		L v M < 0.0001	ref		L v M = 0.2111
Median risk group (M)	≤121.9	54.4	2.09	1.56–2.80	L v H < 0.0001	1.35	0.92–1.98	L v H < 0.0001
High risk group (H)	121.9+	35.5	3.54	2.58–4.86	M v H = 0.0002	3.81	2.46–5.91	M v H < 0.0001
Stage IIIA								
Low risk group (L)	≤60.0	94.6	ref		L v M < 0.0001	ref		L v M < 0.0316
Median risk group (M)	≤90.4	84.3	3.67	2.76–4.87	L v H < 0.0001	2.10	1.36–3.25	L v H < 0.0001
High risk group (H)	90.4+	59.4	11.71	8.66–15.84	M v H < 0.0001	9.07	5.70–14.45	M v H < 0.0001
Stage IIIB								
Low risk group (L)	≤91.0	82.3	ref		L v M < 0.0001	ref		L v M < 0.0001
Median risk group (M)	≤116.9	63.7	2.28	2.09–2.48	L v H < 0.0001	2.72	2.42–3.07	L v H < 0.0001
High risk group (H)	116.9+	36.6	5.06	4.60–5.56	M v H < 0.0001	6.55	5.75–7.47	M v H < 0.0001
Stage IIIC								
Low risk group (L)	≤110.6	63.3	ref		L v M < 0.0001	ref		L v M < 0.0001
Median risk group (M)	≤137.0	35.7	2.10	1.86–2.37	L v H < 0.0001	1.94	1.68–2.24	L v H < 0.0001
High risk group (H)	137.0+	18.2	3.97	3.47–4.55	M v H < 0.0001	4.33	3.68–5.11	M v H < 0.0001
Stage IV								
Low risk group (L)	≤157.2	24.2	ref		L v M < 0.0001	ref		L v M < 0.0001
Median risk group (M)	≤183.3	9.9	1.59	1.49–1.69	L v H < 0.0001	1.87	1.73–2.03	L v H < 0.0001
High risk group (H)	183.3+	3.0	2.79	2.58–3.01	M v H < 0.0001	3.51	3.19–3.88	M v H < 0.0001

*AJCC* American joint committee on cancer, *HR* hazard ratio, *95 % CI* 95 % confident interval, *L* low risk group, *M* median risk group, *H* high risk group

**Fig. 9:**
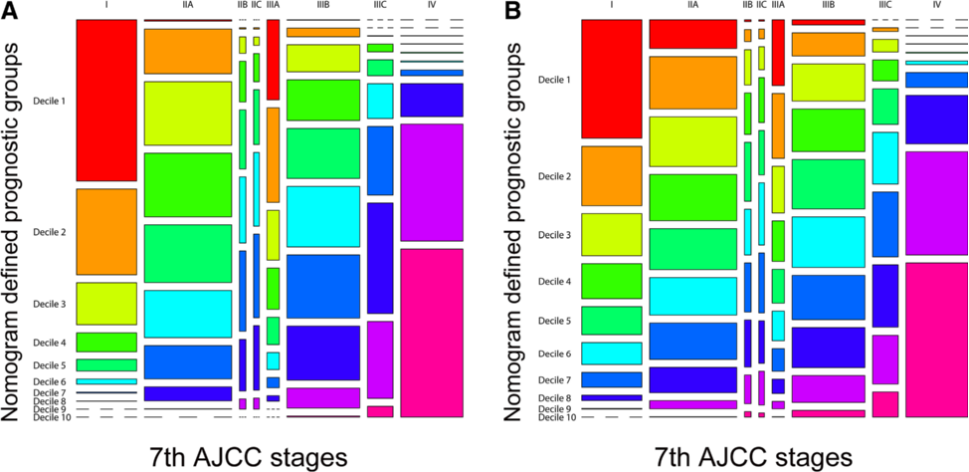
Mosaic plots using the training cohort. **a** CSS, **b** OS. In the mosaic plots, each of the 10 deciles is represented by 1 of 10 consecutive rainbow colors. The area of the individual mosaics represents the relative frequency associated with the column cell. The short segmented lines indicate a frequency of zero. CSS, cancer-specific survival; OS, overall survival; AJCC, American Joint Committee on Cancer

Finally, the mosaic plots demonstrated significant survival heterogeneity within individual AJCC7 substages in contrast to the Nomo stages (Fig. 9). The results offer direct evidence and the underlying frequencies of staging errors in the conventional AJCC staging system.

### Decision curve analysis

**Fig. 10:**
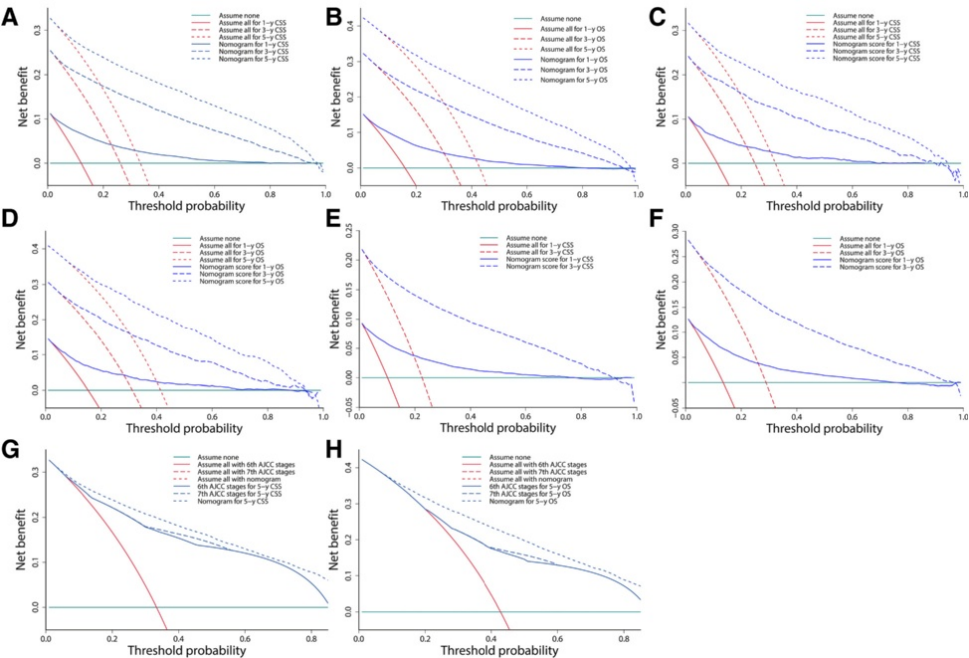
Decision curve analysis of nomograms in different cohorts and model comparisons with AJCC stages. **a ** One-, 3-, and 5-year CSS in the training cohort. **b** One-, 3-, and 5-year OS in the training cohort. **c** One-, 3-, and 5-year CSS in the validation cohort. **d** One-, 3-, and 5-year OS in the validation cohort. **e** One- and 3-year CSS in the test cohort. **f** One- and 3-year OS in the test cohort. **g** Nomogram comparisons of the 6th and 7th versions of the AJCC stages for predicting 5-year CSS in the training cohort. **h** Nomogram comparisons of the 6th and 7th versions of the AJCC stages for predicting 5-year OS in the training cohort. Cyan horizontal lines represent the assumption that events occurred in no patient within a particular timespan. Red lines represent the assumption that events occurred in all patients within the same timespan. Blue lines represent the net benefit of model prediction. The net benefit per patient of each predictive model within a particular timespan was a function of the cohort size with threshold probability, and was computed by addition of the benefit (true positive) and subtraction of the harm (false positive). **g**, **h ** Note that the red “assume all” lines overlap and appear to be a single line for both the nomogram and the 6th- and 7th-version AJCC stages prediction. This occurred because they were constructed using the same cohorts and time points. CSS, cancer-specific survival; OS, overall survival; AJCC, American Joint Committee on Cancer

After addressing the model accuracy, DCA was applied to render clinical validity to the nomograms in the derivation cohort and generalize it to the external cohorts. The results corroborated good clinical applicability of the nomograms in predicting survival of patients with CRC because their ranges of threshold probabilities were wide and practical in all cohorts (Fig. 10). Additional comparisons of model competence were also in favor of the nomograms’ superiority over the conventional AJCC stages because the net benefit for the patients was consistently enhanced (higher lines for model prediction relative to the horizontal lines) when using the nomograms compared with using the TNM stages (Fig. 10).

## Discussion

In the present study, we developed two postoperative nomograms to predict CSS and OS for patients who have undergone CRC resection without neoadjuvant therapy. The nomograms consistently achieved considerable predictive accuracy and appreciable reliability and reproducibility when applied to the derivation and validation cohorts. DCA subsequently demonstrated significant clinical applicability of the nomograms with wide threshold probabilities. In addition, model comparisons and DCA proved that the nomograms outperformed the conventional AJCC stages by stratifying them into three significant prognostic groups and allowing for more robust risk classification (Nomo stages) with an improved net benefit.

Prognostic nomograms are simplified representations of complicated statistical models with elegant graphics [18, 29, 30]. Compared with other predictive models, they are more accurate and comprehensible with user-friendly interfaces, allowing for wide application in clinical practice [18, 29, 30]. A recent systematic review summarized the basic characteristics of more than 16 predictive nomograms for CRC [31]. Although patient definitions, endpoints, and time points are markedly heterogeneous, most of the nomograms have demonstrated improved accuracy. Our study shows some distinctions from those published nomograms, however.

First, no previous studies incorporated both patients with non-metastatic CRC and those with metastatic CRC. Because both non-metastatic and metastatic CRC are continuous representations of systemic tumor biology, exclusion of patients with metastasis may inherit the limitations of the AJCC stages. Second, we used population-based data to derive nomograms for CRC; this may be considered an update and extension of a previously published nomogram that also used SEER data but concentrated on curative stage I to III colonic adenocarcinomas [32]. Population-based data often fail to include detailed data and novel markers such as the CEA concentration [19] and microRNAs [33], which may be helpful to increase model accuracy. However, population-based data are more likely to overcome inconsistency biased by institutional practice [18]. Third, we selected covariates based on the AIC instead of statistical significance (*P* value), allowing for confidence in the robustness of modeling and performance [34]. The *P* values depend not only on the magnitude of the predictors’ effects but also on the sample size. Small data sets are less likely to discriminate small differences, and their use makes it more difficult to reject the null hypothesis. We also used restricted cubic spline functions for continuous variables to avoid unnecessary information loss caused by categorization [23]. Finally, we introduced DCA and proved the clinical validity of our nomograms. High predictive accuracy is not necessarily associated with usefulness in clinical practice. Well-performing models may have limited applicability if the threshold probabilities of the net benefits are impractical, meaning that the new predictive models will be less beneficial than currently available tools and may even be harmful [18, 26].

Our study also produced some novel findings besides the many results consistent with previous studies. Above all, based on the nomograms, we have proposed Nomo stages and efficiently classified stage I to IV CRCs into 10 significant subgroups with a single predictive score. Our nomograms also enable stratification of each AJCC7 substage into three significant risk strata, which has not been achieved by other CRC nomograms. This risk classification and stratification may be very useful for clinicians to identify postoperative patients with high risks associated with intensified follow-up (i.e., patients with high-risk stage I CRC) and select less heterogeneous patients for clinical trials (i.e., patients with high-risk stage II CRC). This also helps to understand the degree of survival heterogeneity in the AJCC stages, which frequently introduces confusion and uncertainty to patient consulting. Note that the optimal thresholds for risk classification and stratification may be individualized, although the thresholds defined by the training cohort still worked well in our external cohorts, which are only intended for relatively strict validation. Additionally and importantly, the sharing of similar contributing predictors is a reflection of apparent correlations between CSS and OS. Some of these predictors are worth noting here. In our models, age had a persistent effect but multiplied from beyond 60 years old. Age is a traditional reference for physical condition, frequency and efficiency of reinforced therapies, thus exerts an accumulated effect on survival. It is reasonable to presume that certain tumor-related factors such as infiltration depth, metastasis, histology, LNR, and LNC are relatively more important predictors than age. They are typical features of tumor development and are closely related to patient death at various but statistically significant levels. The LNC is one of the most controversial among these tumor-related factors. It has been proposed as a quality indicator [6, 35] and is augmented in extended lymphadenectomy, the relevant long-term benefit of which has not been effectively demonstrated because of the absence of prospective clinical trials of extended colonic surgeries [35, 36]. Inadequate LNC assessment is involved in interpretation of stage migration, which is considered a source of survival heterogeneity in patients with CRC, but its influences are limited [14, 35, 36]. Several previous studies classified patients by the 12-node benchmark to derive high- and low-risk subpopulations but achieved inconsistent results, while our results indicate that such classification might be associated with a risk of dichotomizing complex, non-linear effects of LNC on patient survival [37]. Moreover, our analyses indicated that LNC was less superior to LNR, which explains the reduced survival in the patient subset with limited numbers of metastatic nodes. Additionally, the preoperative CEA concentration provides a baseline quantification of the tumor burden and severity of disease. The CEA concentration, with its individualized information and wide application, is due to play a role in the staging of CRC. Next, the effects of racial background may be multifactorial. The lowest prevalence and mortality of CRC are seen in East Asians because of the low prevalence of risk factors such as smoking and obesity in this population [1, 2]. The highest incidence and mortality are seen in black people [2]; this can be ascribed to the lower income, later diagnosis, and less access to high-quality health care in this population [2, 20]. Additionally, marriage makes a prognostic difference [20] and deserves more attention because it may provide compensative mechanisms for improvements in survival. Marital status and ethnicity were introduced to prognostic nomograms for CRC for the first time in the present study. It should also be noted that the nomogram points translated from the models’ coefficients reflect the importance of the variables relative to the presence of the other covariates. They may vary depending on the outcomes measured. Due to the existence of competing risks, the predictive accuracy for OS tended to be lower than that for CSS in our study. However, we chose a Cox proportional hazard model without competing risks because it was easier to interpret, compare, and comprehend [38].

Our study has limitations that deserve attention. Improved model accuracy frequently comes at the cost of increased complexity. The tradeoffs between comprehensiveness and comprehensibility are not easy to balance, and this is a common problem during modeling for nomograms. Considering this, we only selected variables that were clinically important and practical with high reproducibility and low time-varying effects. The nomogram itself is associated with uncertainty. Therefore, we provided 95 % CIs for the c-indexes and calibrations to determine the degree of uncertainty. Because of the shorter follow-up, the c-indexes were slightly higher in the test cohort than in the derivation cohort. However, the time-dependent ROC showed that the predictive AUCs of the nomograms in different cohorts were very close in the same years. Moreover, our nomograms were developed for risk assessment and selection of patients who might benefit from additional interventions after surgery. These interventions may include but are not restricted to adjuvant therapies, strengthened treatments, intensified follow-ups, and motivated patient consulting. Even so, nomograms cannot substitute for clinicians’ judgments or act as exclusive evidence for clinical decision-making. Finally, details regarding tumor deposit, curability of stage IV CRC, and postoperative chemoradiotherapy among the patients in the present study are unknown, placing a limitation on the survival analysis. Incorporation of the new predictors and introduction of competing risk models may further improve model performance [18, 29]. However, this will require new nomograms with different modeling strategies.

## Conclusions

In the present study, the bootstrap-corrected and prospectively validated nomograms were consistently reliable and clinically practical with wide threshold probabilities. Moreover, the nomograms outperformed the conventional AJCC stages by allowing for more robust risk classification and stratifying the AJCC stages with an improved net benefit. However, independent external validations are still required in the future.

## Abbreviations

AIC: akaike information criterion
AJCC: American joint committee on cancer
AUC: area under the receiver-operating characteristic curve
CEA: carcinoembryonic antigen
CI: confident interval
CRC: colorectal cancer
CSS: cancer-specific survival
DCA: decision curve analysis
LNC: lymph node count
LNR: lymph node ratio
OS: overall survival
ROC: receiver-operating characteristic
SEER: surveillance, epidemiology, and end results
